# IL-17: Balancing Protective Immunity and Pathogenesis

**DOI:** 10.1155/2023/3360310

**Published:** 2023-08-12

**Authors:** Lu Sun, Lufei Wang, Bethany B. Moore, Shaoping Zhang, Peng Xiao, Ann M. Decker, Hom-Lay Wang

**Affiliations:** ^1^Department of Periodontics and Oral Medicine, University of Michigan School of Dentistry, Ann Arbor, MI, USA; ^2^Division of Oral and Craniofacial Health Sciences, University of North Carolina at Chapel Hill School of Dentistry, Chapel Hill, NC, USA; ^3^Department of Microbiology and Immunology, University of Michigan School of Medicine, Ann Arbor, MI, USA; ^4^Department of Periodontics, University of Iowa College of Dentistry, Iowa, IA, USA; ^5^Department of Gastroenterology, Sir Run Run Shaw Hospital, Zhejiang University School of Medicine, Hangzhou, China; ^6^Immunological Disease Research Center, Sir Run Run Shaw Hospital, Zhejiang University School of Medicine, Hangzhou, China

## Abstract

The biological role of interleukin 17 (IL-17) has been explored during recent decades and identified as a pivotal player in coordinating innate and adaptive immune responses. Notably, IL-17 functions as a double-edged sword with both destructive and protective immunological roles. While substantial progress has implicated unrestrained IL-17 in a variety of infectious diseases or autoimmune conditions, IL-17 plays an important role in protecting the host against pathogens and maintaining physiological homeostasis. In this review, we describe canonical IL-17 signaling mechanisms promoting neutrophils recruitment, antimicrobial peptide production, and maintaining the epithelium barrier integrity, as well as some noncanonical mechanisms involving IL-17 that elicit protective immunity.

## 1. Introduction

During the last few decades, understanding the pathophysiology of inflammatory diseases has been expanded by the discovery of the existence of different T cell subsets. Effector T helper (Th) cells are derived from naive CD4^+^ T cells triggered by the engagement of T cell receptor (TCR) and costimulatory molecules under the presence of specific cytokines. The main subsets of activated CD4^+^ T cells include specialized Th1/Th2 cells and Treg cells with distinct functional profiles [1, 2]. This aforementioned paradigm was challenged by the discovery of other T cell subsets including T helper 17 (Th17) cells, Th9, Th22, and T follicular helper (Tfh) cells [3–6]. Retinoic acid receptor-related orphan receptor-*γ*t (ROR*γ*t) and signal transducer and activator of transcription 3 (STAT3) are the key transcription factors in activating the differentiation of the program of committed Th17 cells. Transforming growth factor-*β* (TGF-*β*) and proinflammatory cytokines such as interleukin 6 (IL-6) are critical cytokines for murine Th17 cell differentiation [7]. The signature cytokine, IL-17, of Th17 cells has pleiotropic roles targeting both nonhematopoietic cells, including fibroblasts and epithelial cells, and hematopoietic cells. IL-17 has emerged as having dichotomous roles due to both destructive and protective effects in various diseases, especially infectious diseases and autoimmune diseases. This review summarizes the basic pathogenic roles of IL-17 and particularly focuses on the protective role of IL-17 responses.

## 2. IL-17 Cytokine and Signaling

**Figure 1:**
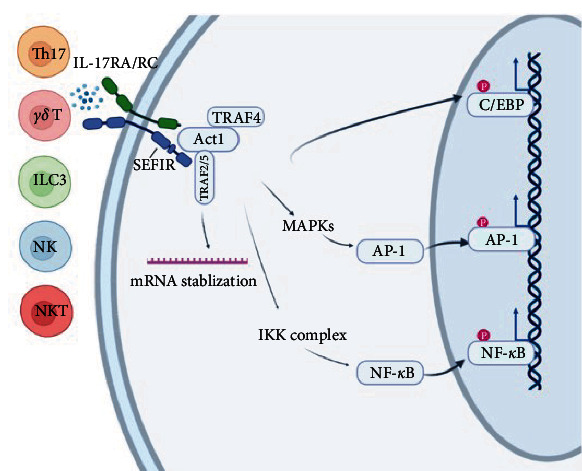
Schematic representation of the IL-17 signaling pathway: transduction and amplification. The IL-17RA and IL-17RC subunits bind to IL-17A, IL-17F, and IL-17AF ligands. The intracellular domains interact with adaptor Act1. Act1 additionally contains a TRAF-binding site that enables association with TRAF family proteins. Engagement with TRAF6 drives activation of the classical NF-*κ*B, MAPK pathway. Act1 can also engage other TRAF family proteins to promote a post-transcriptional mRNA stabilization pathway. Th17, T helper-17 cells; *γδ* T cells, gamma delta T cells; ILC, innate lymphoid cells; NKT nature killer T cells; TRAF, TNF-receptor associated factor; MAPK, mitogen-activated protein kinase; NF-*κ*B, nuclear factor *κ*B; AP-1, activator protein; C/EBP, CCAAT enhancer-binding protein.

IL-17, the hallmark cytokine of Th17, was cloned and named as CTLA8 in 1993 [8]. The IL-17 cytokine family consists of six members based on sequence homology, called IL17-A, IL17-B, IL-17C, IL-17D, IL-17E (also known as IL-25), and IL-17F [9, 10]. IL-17A and IL-17F can form either heterodimers or homodimers, as they have closely related expression patterns and sequences [11]. IL-17A (also commonly called IL-17) has been the most studied member of the IL-17 cytokine family. Besides Th17 cells, various innate and acquired immune cells are also capable of producing IL-17 (Figure 1). The main non-Th17 cellular sources that can produce IL-17 cytokines include type 3 innate lymphoid cells (ILC3), *γδ* T cells, CD8^+^ T cells, and natural killer cells [12–16]. It has also been reported that neutrophils can produce IL-17 after stimulation with LPS or recombinant IL-6 and IL-23 [17, 18]. There are five different subunits of IL-17 receptors, IL-17RA, IL-17RB, IL-17 RC, IL-17 RD, and IL-17 RE [14]. IL-17 receptors are expressed widely in different cells and tissue types. IL-17 signals via a heterodimeric IL-17RA/IL-17RC complex [19, 20].

A conserved cytoplasmic motif known as the “similar expression of fibroblast growth factor and IL-17R” (SEFIR) domain was identified within the members of the IL17R family, which is closely related to Toll/interleukin-1 receptor (TIR) domains expressed in Toll-like receptor (TLR) and IL-1R family members [10, 21]. Adaptor for IL-17 receptor (Act1) (nuclear factor-kappa B (NF-*κ*B) activator 1), also known as CIKS encoded by the gene *TRAF3IP2* (TRAF3 interacting protein 2), also contains the SEFIR domain and is an indispensable component in the IL-17 signaling pathway [22, 23]. IL-17R/Act1 signaling activation is mediated through SEFIR domain–SEFIR domain interaction that recruits tumor necrosis factor-R-associated factor 6 (TRAF6), a critical upstream activator for transcription factor NF-*κ*B, AP-1 (activator protein-1), and C/EBP (CCAAT/enhancer-binding protein) [10, 24–26], all of which induce a panel of inflammatory mediators to respond to pathogens (Figure 1). While IL-17-mediated responses involved in infection and inflammatory diseases, it is important to note that signaling through IL-17/IL-17R also plays a protective role through multiple mechanisms, such as regulating the recruitment and granulopoiesis of neutrophils, producing antimicrobial peptides and maintaining barrier integrity [27, 28]. The regulation of pathologic versus protective IL-17 responses involves a complex interplay of several factors. Transcriptional factors, including ROR*γ*t, STAT3, IRF4, and FoxP3, play critical roles in the differentiation, function, and balance of Th17 cells and Tregs, thereby influencing the outcome of IL-17-mediated immune responses. Specifically, ROR*γ*t is considered the master transcription factor for IL-17 production and promotes the differentiation of naïve CD4^+^ T cells into Th17 cells, which produce IL-17. Activated STAT3 plays a crucial role in promoting IL-17 production by enhancing its transctiption and IRF4 interacts with ROR*γ*t and collaborates in the regulation of IL-17 expression by binding to the IL-17 gene promoter. On the contrary, FoxP3 is a transcription factor that plays a crucial role in the development and function of regulatory T cells (Tregs). Dysregulation of these factors can contribute to the development of inflammatory diseases by either excessive IL-17 production or impaired regulatory mechanisms. Furthermore, the cytokine milieu and the local microenvironment, such as a balance between immune cell types, at the site of infection or inflammation also influence the balance between pathologic and protective IL-17 responses. Understanding these regulatory mechanisms is crucial for the development of targeted therapies that can modulate IL-17 responses in a beneficial way, promoting host defense while minimizing tissue damage and chronic inflammation in various infectious and inflammatory diseases.

## 3. Immunopathogenesis of IL-17

Even though IL-17 is produced in response to most infections, there is convincing evidence suggesting improperly regulated IL-17 and other Th17 cytokines can contribute to the pathogenesis of a variety of diseases [29]. Excessive IL-17 can be detrimental for many human inflammatory and autoimmune diseases including psoriasis, arthritis, Sjogren's disease, and inflammatory bowel disease (IBD) [30–35] (Table 1). In rheumatoid arthritis (RA), for example, IL-17 induced proinflammatory pathogenesis partially by activating osteoclastogenesis that is closely associated with bone resorption in RA patients [36] (Table 1). Periodontitis is a common chronic inflammatory disease caused by microbial infection in the susceptible hosts and it has been documented that IL-17 plays both protective and destructive roles in the progression of periodontitis [37, 38]. Some studies have found that IL-17 dominated the inflammatory network associated with periodontitis traits [39, 40] (Table 1), which indicated that abnormal inflammatory responses induced by IL-17 may cause tissue damage. One potential mechanism is that IL-17 is able to amplify inflammation through excessive neutrophil recruitment by enhancing proinflammatory cytokine/chemokine production, which results in further osteoclast activation and bone resorption [41]. However, emerging evidence demonstrates the role of anti-inflammatory cytokines in regulating IL-17. For instance, IL-10 plays a key role in limiting IL-17-mediated pathology [39]. Furthermore, fibroblasts are one of the most abundant cell types that contribute to the formation of connective tissue. IL-17 not only mediates fibroblast proliferation [42] (Table 1), but also induces fibroblast cells to secrete the matrix metalloproteinase-1 (MMP-1) and MMP-3 causing connective tissue destruction [43]. It is also documented that the presence of bacterial dysbiosis and increased microbial load may be accompanied by hyperproduction of IL-17 in chronic and leukocyte adhesion deficiency I (LAD-I) periodontitis [44]. Notably, IL-17 signaling was critical for acute lung injury of influenza infection and *S. pneumoniae* coinfection with influenza virus elicits IL-17A response causing inflammation in the nasopharynx [45, 46] (Table 1). Targeting IL-17RA signaling or IL-17A could potentially be a therapeutic strategy to mitigate immunopathology associated with sever influenza infections.

IL-6 is both a signaling mediator that prominently contributes to maintenance of the Th17-cell population and a known pro-inflammatory target in downstream IL-17 signaling. Importantly, the IL-17-mediated positive feedback loop of IL-6 signaling through NF-*κ*B and STAT3 contributes to enhanced autoimmune encephalomyelitis (EAE), whereas neutralizing IL-17 disrupts IL-17-sustaining, IL-6 self-reinforcing loop at the sites of inflammation [47] (Table 1). Regarding digestive disease, there is increased IL-17 expression and number of IL-17-producing T cells in the inflamed mucosa of active IBD patients [32, 48]. Furthermore, genome-wide association studies (GWAS) have identified several Th17/IL-17-associated genetic variants in Crohn's disease and ulcerative colitis patients [49]. There is a clear need to understand how those genetic variants integrate with cells, microbes, and even metabolites in the intestinal microenvironment. Additionally, a recent study demonstrates that the Th17/ROR*γ*t^+^ regulatory T cell balance driven by IBD microbiota is reversible by a defined microbiota transplant in a set of gnotobiotic mouse experiments [50]. The interplay between Th17 cells and Treg cells in the context of intestinal microbiota engages in an extensive bidirectional communication. IL-10 produced by Tregs promotes immune homeostasis whereas expansion of Th17 cells may alter the configuration of the gut microbiome. Furthermore, intestinal microenvironmental factors, such as diet and antibiotic use, may lead to microbiome disturbance. However, the microbiome plays decisive roles in the training and shaping of the host immune system. The crosstalk between perturbations of the gut microbiome and immune dysregulation may finally lead to an inflammatory disorder of the gastrointestinal tract [51]. The studies to identify these alternations and understand them in complex networks may have either diagnostic or therapeutic potential. Indeed, insights into IL-17 studies have spurred efforts to explore and test targeted therapies via different clinical trials with IL-17 inhibitors. Psoriasis is a T cell-mediated inflammatory systemic disease that is characterized by proliferating keratinocytes and erythematous plaques on the skin. The clinical studies of Bimekizumab and Secukinumab have shown the remarkable efficacy of IL-17 inhibition for the treatment of plaque psoriasis [52, 53]. Moreover, IL-17 antagonist netakimab is effective and safe in the treatment of cytokine release syndrome in COVID-19 [54]. Collectively, emerging evidence indicates IL-17 is a key mediator in inflammatory pathogenesis but the mechanisms are complex and await further elucidation.

## 4. IL-17 Canonical Protective Effects

### 4.1. IL-17 Increases the Generation and Recruitment of Neutrophils

**Figure 2:**
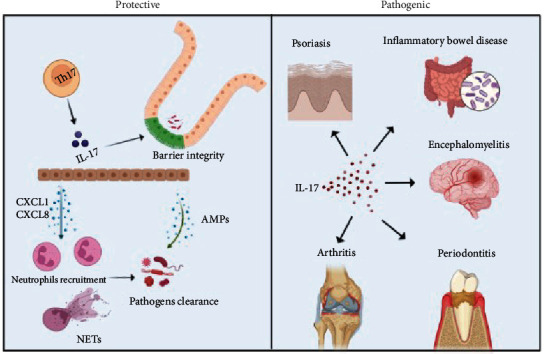
Role of IL-17 in protective immunity versus immunopathology. Protective immunity: IL-17 activates the production of chemokines for neutrophil recruitment and triggers neutrophil extracellular traps (NETs) for pathogen clearance. IL-17 promotes the production of antimicrobial peptides (AMPs) with antibacterial properties through skin or mucosal surfaces. IL-17 also enhances epithelial barrier function to prevent dissemination of pathogens to amplify immune response. Immunopathology: IL-17 mediates tissue inflammation and damage that leads to different inflammatory and autoimmune diseases.

While the presence of IL-17 has been implicated in inflammatory pathogenesis, it exerts protective functions in clearing pathogens and maintaining tissue homeostasis through diverse mechanisms that have also been documented. The most well-known function of IL-17 is its ability to initiate an inflammatory response inducing neutrophil-specific chemokines [(CXCL1, CXCL2, CXCL5, and IL-8) that attract neutrophils from the bloodstream to sites of infection, the expression of adhesion molecules to facilitate the firm attachment and extravasation [10], and granulocyte colony-stimulating factors (G-CSF) that promotes neutrophil generation and migration to the site of infection at surfaces of the skin and mucosa [55]. IL-17 was also reported to induce the expression of granulocyte-macrophage colony-stimulating factor (GM-CSF) in NK cells [56]. Additionally, induced G-CSF and GM-CSF from IL-17 will in turn enhance the expansion and survival of neutrophils [57]. Furthermore, IL-17 plays a role in neutrophil recruitment in limiting pathogens [58–60] (Figure 2). Disruption of IL-17 signaling resulted in bacterial dysbiosis accompanied by earlier autoimmune disease onset in the gut and worsened severity associated with increased G-CSF expression in the intestine and systemic GM-CSF expression in one study [61]. These data might be interpreted as showing neutrophils serve critical protective roles in host defense processes. It has also been reported that IL-17RA-deletion abrogated the increase of splenic neutrophil progenitors [62] and IL-17 signaling played a nonredundant role in neutrophil recruitment in human lung tissue through elaborated G-CSF [63]. Moreover, IL-17RA-deficient mice exhibited increased susceptibility to additional pathogens due to a lack of neutrophil recruitment [64–67].

In addition, IL-17 can synergistically raise IL-1*β*-mediated cellular mRNA induction and protein release of IL-8 via activation of AP-1 and NF-*κ*B [68]. IL-1*β* can also functionally synergize to enhance CCL20 production in human gingival fibroblasts to recruit Th17 cells [69]. Thus, IL-17 can cooperate with IL-1*β* to promote a Th17 laden environment, which in turn may cause a protective situation. Apart from upregulating chemokine expression in epithelial and endothelial cells, the formation of the IL-17 signalosome driven by IL-17-induced dimerization of IL-17RA potentiates CXCL1 mRNA expression in keratinocytes [70] and CXCL1 recruits neutrophils. Furthermore, IL-17 also induces MAPK activation and prolongs the half-life of CXCL1 mRNA [71] and regulates the stability of CXCL1 mRNA transcripts [72]. Zhang et al. [73] further demonstrated that IL-17 potentiates its immunosuppressive effects through tumor-associated neutrophil recruitment and pathogen clearance by neutrophil extracellular traps (NETs). NETosis is increased in severe COVID-19 patients [74] and a potential mechanism that has been suggested is that the cytokine storm might be perpetuated by IL17-induced systemic NETs. Altogether, these studies point out that IL-17 is a key component for neutrophil homeostasis, which is fine-tuned by a balance among granulopoiesis, extravasation of neutrophils into local infected sites, as well as prolonged longevity of neutrophil-specific chemokines.

### 4.2. IL-17 Promotes the Production of Antimicrobial Peptides

The protective mechanisms of IL-17-mediated immunity are not only limited to neutrophil orchestration but also related to antimicrobial peptide (AMP) production, such as S100 proteins, cathelicidin (LL-37 in humans and mCRAMP in mice), *β*-defensins, C-type lectins, and lactoferrins to clear pathogens (Figure 2). AMPs are well recognized as important proteins in innate immunity, especially on the skin and mucosal surface. Strikingly, the host defense peptide cathelicidin is a potentiator for Th17 differentiation and mice lacking cathelicidin cannot increase IL-17 production in response to inflammation [75]. *β*-Defensins are key components of innate immunity, which directly kill or inhibit the growth of some Gram-positive as well as Gram-negative microorganisms. IL-17 enhances antimicrobial peptides produced by human keratinocytes or epithelial cells [76, 77] and IL-17 acts synergistically with IL-22 produced by ILC3s to induce AMP secretion by epithelial cells, such as *β*-defensin 2, *β*-defensin 3, and lipocalins, which play indispensable roles in limiting dissemination of pathogens [78, 79]. Given the synergistic effect of IL-17 and IL-22, it is not surprising that the IL-17/IL-22 alliance functions as an essential component of mucosal immunity to pathogens. TNF-*α* can have an additional synergistic effect with IL-17 to increase the production of AMPs, such as *β*-defensin 2 and S100A7 by keratinocytes [80, 81]. In the past decade, murine studies have provided clues to elucidate the complexity of the intestinal microbiota and the host defense against microbiota to maintain mucosal barrier function and homeostasis through complicated molecular mechanisms. Ivanov et al. [82] reported that the presence of segmented filamentous bacteria (SFB), a single commensal microbe, is sufficient to induce IL-17-producing cells in the small intestine and colonization of SFB reduces the growth of the intestinal pathogen *Citrobacter rodentium* and correlated with antimicrobial peptide secretion. However, the induction of the Th17 population not only depends on the intestinal microbiota, but also depends on constant exposure of the intestine to diet and metabolism as highlighted by multiple studies with distinct metabolic mechanisms impacting different Th17 cell phenotypes [83]. The mechanisms that regulate heterogenous nonpathogenic Th17 cells (npTh17) and pathogenic Th17 cells (pTh17) were further dissected by single-cell ATAC-seq integrated with single-cell RNA-seq showing differences in the chromatin landscape of each [84]. Moreover, IL-17-induced antimicrobial protein regenerating family member 3Alpha (REG3A) in keratinocytes can promote skin proliferation after injury [85]. Taken together, the mounting evidence suggests that another hallmark protective function of IL-17 is stimulating AMP production. However, further studies are needed to fully unravel the regulatory mechanisms surrounding the role of IL-17 in AMP production.

### 4.3. IL-17 Maintaining Barrier Integrity

The fundamental role of the epithelium of the gastrointestinal tract and skin, which combined are the largest human body surface area exposed to the external environment, is developing barrier integrity to resist diverse hostile pathogens. Several diseases have been associated with compromised epithelial barrier function, such as IBD, psoriasis, and atopic dermatitis [86, 87]. A plethora of past studies have implicated IL-17-producing cells or IL-17's role in maintaining homeostasis of barrier integrity and preventing pathogens from invasion [12] (Figure 2). Neutralization of IL-17 aggravates the development of dextran sulfate sodium- (DSS-) induced colitis in mice due to downregulated claudin expression resulting from IL-17 neutralization, resulting in decreased or even compromised mucosal barrier integrity [88]. *γδ* T cells have differential TCR expression and distinct functions such as producing either IFN*γ* or IL-17 [89], which could alter barrier integrity. Interestingly, IL-17 also regulates the tight junction protein occludin during epithelial injury, and the protective effects of IL-17 produced by *γδ* T cells, independent of IL-23 signaling, remains intact in a DSS-induced colitis model [90]. Moreover, inhibition of IL-17 signaling exacerbates colitis that was associated with severe intestinal epithelial barrier dysfunction [91]. These studies are a strong endorsement of the protective effects of IL-17 in forming and maintaining the intestinal mucosa fence. Loss of epithelial barrier function could result in the dissemination of pathogens or commensal bacteria allowing easy access to macrophages and dendritic cells residing below the mucosal barrier, which in turn amplifies the subsequent innate and adaptive immune activation and causes disease development.

Epithelial integrity can also be enhanced by inducing tight junction correlated proteins, such as upregulating claudin gene expression, through the IL-17-mediated ERK MAPK pathway [92]. Kallikrein 1 expression can also be driven by IL-17 in renal epithelial cells to confer protection against *Candida albicans* dissemination and expression of kallikrein is impaired in IL-17RA^−/−^ mice following *C. albicans* infection [93]. Taken together, the commensal organisms or pathogens from the lumen can be kept from passing through tight junction proteins between epithelial cells by IL-17-mediated barrier tightening. It is true that IL-17 also plays a pathogenic role during protective surveillance, thus leading to the paradox of IL-17; tissue-damaging potential is weighed against its protective role in maintaining barrier integrity [90, 94]. Further work will be required to unravel more about the regulation of IL-17 in epithelial barrier function as well as the regulation of IL-17 production from either Th17 cells or other cell types.

## 5. IL-17 Noncanonical Protective Effects

IL-17 has blossomed during the past 30 years and has gained much attention for context-dependent roles in switching between friend and foe. This review largely highlights the beneficial aspects and it is evident that IL-17/IL-17R signaling results in neutrophils recruitment, AMP secretion, and tight junction maintenance.

In addition to the mechanisms mentioned above, IL-17 signaling can also affect T and B cell functions. Act1 is necessary for IL-17-mediated inflammatory responses and functions as a negative regulator in T and B cells via direct inhibition of STAT3 [95]. Majumder et al. [96] recently found a novel role of IL-17 in driving the activation of fibroblastic reticular cells in secondary lymphoid organs through metabolic reprograming, which potentiates the proliferation and survival of these cells as well as promotes B cell responses. Additionally, IL-17 is also known to govern hypoxic adaptation of injured epithelium. [97]. Moreover, proper control of IL-17 might decrease short-term memory deficit and delay mild cognitive impairment in Alzheimer's disease [98, 99]. More research efforts are needed to explore how IL-17 controls central nervous system autoimmunity as well as regulates neuron communication before clinical trials for therapy can be designed.

Finally, the compelling future direction of IL-17 research will investigate proper brakes to control the level and capacity of IL-17 signaling. Future studies may unveil fine-tuned mechanisms for regulating inflammation and maintaining successful immunity with minimal immunopathology. Furthermore, gaining an in-depth understanding of these mechanisms may facilitate discovering and providing new therapeutic drugs. There is no doubt that more context- and tissue-dependent functions of IL-17 in human diseases and homeostasis will be revealed in the following decade.

## Figures and Tables

**Table 1:** IL-17 pathway in autoimmune and inflammatory diseases.

Inflammatory/autoimmune diseases	Evidence of role for IL-17 pathway in different diseases	Refs.
Arthritis	IL-17A^+^CD8^+^ T cells were predominantly TCR*αβ*+ and their frequencies were increased in the synovial fluid of patients with established arthritis. IL-17 in synovial fluids from patients with rheumatoid arthritis is a potent stimulator of osteoclastogenesis.	[33, 36]
Sjogren's disease	A significant increase of IL-17 expressing cells in salivary glands involved in the onset and progression of Sjogren's disease.	[34]
Inflammatory bowel disease	Pathogenic CXCR6^+^ Th17 populations are induced in autoimmunity.	[30]
Periodontitis	IL-17 dominated an inflammatory network characteristic of periodontitis, and IL-10 dampens this excessive IL-17-mediated periodontitis trait. Homeostatic IL-17-TRAF3IP2-neutrophil axis underpinning host defense against a keystone periodontal pathogen.	[39, 40]
Encephalomyelitis	IL-17-mediated positive feedback loop of IL-6 signaling through NF-*κ*B and STAT3 contributes to enhanced autoimmune encephalomyelitis.	[47]
Virus associated inflammation	Bone marrow-derived IL-17A is required for the development of pneumonitis. IL-17 signaling is critical for lung immunopathology associated with virus infection.	[42, 45, 46]

## References

[1] Mosmann T. R., Cherwinski H., Bond M. W., Giedlin M. A., Coffman R. L. (1986). Two types of murine helper T cell clones. I. definition according to profiles of lymphokine activities and secreted proteins. *The Journal of Immunology*.

[2] Sakaguchi S., Kassiotis G., Liston A. (2011). Regulatory T cells: history and perspective. *Regulatory T Cells*.

[3] Harrington L. E., Hatton R. D., Mangan P. R. (2005). Interleukin 17-producing CD4^+^ effector T cells develop via a lineage distinct from the T helper type 1 and 2 lineages. *Nature Immunology*.

[4] Dardalhon V., Awasthi A., Kwon H. (2008). IL-4 inhibits TGF-*β*-induced Foxp3^+^ T cells and, together with TGF-*β*, generates IL-9^+^ IL-10^+^ Foxp3− effector T cells. *Nature Immunology*.

[5] Duhen T., Geiger R., Jarrossay D., Lanzavecchia A., Sallusto F. (2009). Production of interleukin 22 but not interleukin 17 by a subset of human skin-homing memory T cells. *Nature Immunology*.

[6] Crotty S. (2014). T follicular helper cell differentiation, function, and roles in disease. *Immunity*.

[7] Bettelli E., Carrier Y., Gao W. (2006). Reciprocal developmental pathways for the generation of pathogenic effector T_H_17 and regulatory T cells. *Nature*.

[8] Rouvier E., Luciani M. F., Mattéi M. G., Denizot F., Golstein P. (1993). CTLA-8, cloned from an activated T cell, bearing AU-rich messenger RNA instability sequences, and homologous to a herpesvirus saimiri gene. *Journal of immunology*.

[9] Amatya N., Garg A. V., Gaffen S. L. (2017). IL-17 signaling: the yin and the yang. *Trends in Immunology*.

[10] Gaffen S. L. (2009). Structure and signalling in the IL-17 receptor family. *Nature Reviews Immunology*.

[11] McGeachy M. J., Cua D. J., Gaffen S. L. (2019). The IL-17 family of cytokines in health and disease. *Immunity*.

[12] Cua D. J., Tato C. M. (2010). Innate IL-17-producing cells: the sentinels of the immune system. *Nature Reviews Immunology*.

[13] Sutton C. E., Mielke L. A., Mills K. H. G. (2012). IL-17-producing *γδ* T cells and innate lymphoid cells. *European Journal of Immunology*.

[14] Patel D. D., Kuchroo V. K. (2015). Th17 cell pathway in human immunity: lessons from genetics and therapeutic interventions. *Immunity*.

[15] Artis D., Spits H. (2015). The biology of innate lymphoid cells. *Nature*.

[16] Kolls J. K., Lindén A. (2004). Interleukin-17 family members and inflammation. *Immunity*.

[17] Ferretti S., Bonneau O., Dubois G. R., Jones C. E., Trifilieff A. (2003). IL-17, produced by lymphocytes and neutrophils, is necessary for lipopolysaccharide-induced airway neutrophilia: IL-15 as a possible trigger. *The Journal of Immunology*.

[18] Taylor P. R., Roy S., Leal S. M. (2014). Activation of neutrophils by autocrine IL-17A–IL-17RC interactions during fungal infection is regulated by IL-6, IL-23, ROR*γ*t and dectin-2. *Nature Immunology*.

[19] Toy D., Kugler D., Wolfson M. (2006). Cutting edge: interleukin 17 signals through a heteromeric receptor complex. *The Journal of Immunology*.

[20] Song X., Zhu S., Shi P. (2011). IL-17RE is the functional receptor for IL-17C and mediates mucosal immunity to infection with intestinal pathogens. *Nature Immunology*.

[21] Novatchkova M., Leibbrandt A., Werzowa J., Neubüser A., Eisenhaber F. (2003). The STIR-domain superfamily in signal transduction, development and immunity. *Trends in Biochemical Sciences*.

[22] Mellett M., Atzei P., Bergin R. (2015). Orphan receptor IL-17RD regulates Toll-like receptor signalling via SEFIR/TIR interactions. *Nature Communications*.

[23] Boisson B., Wang C., Pedergnana V. (2013). An *ACT1* mutation selectively abolishes interleukin-17 responses in humans with chronic mucocutaneous candidiasis. *Immunity*.

[24] Ruddy M. J., Wong G. C., Liu X. K. (2004). Functional cooperation between Interleukin-17 and tumor necrosis Factor-*α* is mediated by CCAAT/enhancer-binding protein family members. *Journal of Biological Chemistry*.

[25] Chen J., Liao M.-Y., Gao X.-L. (2013). IL-17A induces pro-inflammatory cytokines production in macrophages via MAPKinases, NF-*κ*B and AP-1. *Cellular Physiology and Biochemistry*.

[26] Bulek K., Liu C., Swaidani S. (2011). The inducible kinase IKKi is required for IL-17-dependent signaling associated with neutrophilia and pulmonary inflammation. *Nature Immunology*.

[27] Abusleme L., Moutsopoulos N. M. (2017). IL-17: overview and role in oral immunity and microbiome. *Oral Diseases*.

[28] Conti H. R., Shen F., Nayyar N. (2009). Th17 cells and IL-17 receptor signaling are essential for mucosal host defense against oral candidiasis. *Journal of Experimental Medicine*.

[29] Mills K. H. G. (2023). IL-17 and IL-17-producing cells in protection versus pathology. *Nature Review Immunology*.

[30] Schnell A., Huang L., Singer M. (2021). Stem-like intestinal Th17 cells give rise to pathogenic effector T cells during autoimmunity. *Cell*.

[31] Schmitt H., Neurath M. F., Atreya R. (2021). Role of the IL23/IL17 pathway in Crohn’s Disease. *Frontiers in Immunology*.

[32] Moschen A. R., Tilg H., Raine T. (2019). IL-12, IL-23 and IL-17 in IBD: immunobiology and therapeutic targeting.. *Nature Reviews Gastroenterology & Hepatology*.

[33] Steel K. J. A., Srenathan U., Ridley M. (2022). Polyfunctional, proinflammatory, tissue-resident memory phenotype and function of synovial interleukin-17A+CD8+ T cells in psoriatic arthritis. *Arthritis & Rheumatology*.

[34] Hwang S.-H., Woo J. S., Moon J. (2021). IL-17 and CCR9^+^*α*4*β*7^−^ Th17 cells promote salivary gland inflammation, dysfunction, and cell death in Sjögren’s Syndrome. *Frontiers in Immunology*.

[35] Zwicky P., Unger S., Becher B. (2019). Targeting interleukin-17 in chronic inflammatory disease: a clinical perspective. *Journal of Experimental Medicine*.

[36] Kotake S., Udagawa N., Takahashi N. (1999). IL-17 in synovial fluids from patients with rheumatoid arthritis is a potent stimulator of osteoclastogenesis. *Journal of Clinical Investigation*.

[37] Huang N., Dong H., Luo Y., Shao B. (2021). Th17 cells in periodontitis and its regulation by A20. *Frontiers in Immunology*.

[38] Feng Y., Chen Z., Tu S.-Q. (2022). Role of Interleukin-17A in the pathomechanisms of periodontitis and related systemic chronic inflammatory diseases. *Frontiers in Immunology*.

[39] Sun L., Girnary M., Wang L. (2020). IL-10 dampens and IL-17-mediated periodontitis-associated inflammatory network. *Journal of Immunology*.

[40] Zhang J., Sun L., Withanage M. H. H. (2023). TRAF3IP2-IL-17 axis strengthens the gingival defense against pathoges. *Journal of Dental Research*.

[41] Moutsopoulos N. M., Chalmers N. I., Barb J. J. (2015). Subgingival microbial communities in Leukocyte Adhesion Deficiency and their relationship with local immunopathology. *PLOS Pathogens*.

[42] Zhou X., Loomis-King H., Gurczynski S. J. (2016). Bone marrow transplantation alters lung antigen-presenting cells to promote T_H_17 response and the development of pneumonitis and fibrosis following gammaherpesvirus infection. *Mucosal Immunology*.

[43] van Hamburg J. P., Asmawidjaja P. S., Davelaar N. (2011). Th17 cells, but not Th1 cells, from patients with early rheumatoid arthritis are potent inducers of matrix metalloproteinases and proinflammatory cytokines upon synovial fibroblast interaction, including autocrine interleukin-17A production. *Arthritis & Rheumatism*.

[44] Eskan M. A., Jotwani R., Abe T. (2012). The leukocyte integrin antagonist Del-1 inhibits IL-17-mediated inflammatory bone loss. *Nature Immunology*.

[45] Crowe C. R., Chen K., Pociask D. A. (2009). Critical role of IL-17RA in immunopathology of influenza infection. *Journal of Immunology*.

[46] Ambigapathy G., Schmit T., Mathur R. K. (2019). Double-edged role of interleukin 17A in *Streptococcus pneumoniae* pathogenesis during influenza virus coinfection. *The Journal of Infectious Disease*.

[47] Ogura H., Murakami M., Okuyama Y. (2008). Interleukin-17 promotes autoimmunity by triggering a positive-feedback loop via interleukin-6 induction. *Immunity*.

[48] Noviello D., Mager R., Roda G., Borroni R. G., Fiorino G., Vetrano S. (2021). The IL23-IL17 immune axis in the treatment of ulcerative colitis: successes, defeats, and ongoing challenges. *Frontiers in Immunology*.

[49] Khor B., Gardet A., Xavier R. J. (2011). Genetics and pathogenesis of inflammatory bowel disease. *Nature*.

[50] Britton G. J., Contijoch E. J., Spindler M. P. (2020). Defined microbiota transplant restores Th17/ROR*γ*t^+^ regulatory T cell balance in mice colonized with inflammatory bowel disease microbiotas. *Proceedings of the National Academy of Sciences*.

[51] Zheng D., Liwinski T., Elinav E. (2020). Interaction between microbiota and immunity in health and disease. *Cell Research*.

[52] Reich K., Warren R. B., Lebwohl M. (2021). Bimekizumab versus secukinumab in plaque psoriasis. *The New England Journal of Medicine*.

[53] Warren R. B., Blauvelt A., Bagel J. (2021). Bimekizumab versus adalimumab in plaque psoriasis. *The New England Journal of Medicine*.

[54] Maslennikov R., Ivashkin V., Vasilieva E. (2021). Interleukin 17 antagonist netakimab is effective and safe in the new coronavirus infection (COVID-19). *European Cytokine Network*.

[55] Gurczynski S. J., Nathani N., Warheit-Niemi H. I. (2019). CCR2 mediates increased susceptibility ot post-H1N1 bacterial pneumonia by limiting dendritic cell induction of IL-17. *Mucosal Immunology*.

[56] Bar E., Whitney P. G., Moor K., Reis e Sousa C., LeibundGut-Landmann S. (2014). IL-17 regulates systemic fungal immunity by controlling the functional competence of NK cells. *Immunity*.

[57] Parsonage G., Filer A., Bik M. (2008). Prolonged, granulocyte-macrophage colony-stimulating factor-dependent, neutrophil survival following rheumatoid synovial fibroblast activation by IL-17 and TNFalpha. *Arthritis Research & Therapy*.

[58] McGinley A. M., Sutton C. E., Edwards S. C. (2020). Interleukin-17A serves a priming role in autoimmunity by recruiting IL-1*β*-producing myeloid cells that promote pathogenic T cells. *Immunity*.

[59] Borkner L., Curham L. M., Wilk M. M., Moran B., Mills K. H. G. (2021). IL-17 mediates protective immunity against nasal infection with *Bordetella pertussis* by mobilizing neutrophils, especially Siglec-F^+^ neutrophils. *Mucosal immunology*.

[60] Tang J., Li J., Pan J. (2023). The protective role of interleukin 17A in *Acinetobacter baumannii* pneumonia is associated with *Candida albicans* in the airway. *Infection and Immunity*.

[61] Kumar P., Monin L., Castillo P. (2016). Intestinal interleukin-17 receptor signaling mediates reciprocal control of the gut microbiota and autoimmune inflammation. *Immunity*.

[62] Ye P., Rodriguez F. H., Kanaly S. (2001). Requirement of interleukin 17 receptor signaling for lung Cxc chemokine and granulocyte colony-stimulating factor expression, neutrophil recruitment, and host defense. *Journal of Experimental Medicine*.

[63] McAllister F., Henry A., Kreindler J. L. (2005). Role of IL-17A, IL-17F, and the IL-17 receptor in regulating growth-related oncogene-alpha and granulocyte colony-stimulating factor in bronchial epithelium: implications for airway inflammation in cystic fibrosis. *Journal of Immunology*.

[64] Ouyang W., Kolls J. K., Zheng Y. (2008). The biological functions of T helper 17 cell effector cytokines in inflammation. *Immunity*.

[65] Chung D. R., Kasper D. L., Panzo R. J. (2003). CD4^+^ T cells mediate abscess formation in intra-abdominal sepsis by an IL-17-dependent mechanism. *Journal of Immunology*.

[66] Kelly M. N., Kolls J. K., Happel K. (2005). Interleukin-17/interleukin-17 receptor-mediated signaling is important for generation of an optimal polymorphonuclear response against *Toxoplasma gondii* infection. *Infection and Immunity*.

[67] Huang W., Na L., Fidel P. L., Schwarzenberger P. (2004). Requirement of interleukin-17A for systemic anti-Candida albicans host defense in mice. *The Journal of Infectious Diseases*.

[68] Dragon S., Rahman M. S., Yang J., Unruh H., Halayko A. J., Gounni A. S. (2007). IL-17 enhances IL-1beta-mediated CXCL-8 release from human airway smooth muscle cells. *American Journal of Physiology-Lung Cellular and Molecular Physiology*.

[69] Hirota K., Yoshitomi H., Hashimoto M. (2007). Preferential recruitment of CCR6-expressing Th17 cells to inflamed joints via CCL20 in rheumatoid arthritis and its animal model. *Journal of Experimental Medicine*.

[70] Goepfert A., Barske C., Lehmann S. (2022). IL-17-induced dimerization of IL-17RA drives the formation of the IL-17 signalosome to potentiate signaling. *Cell Reports*.

[71] Sun D., Novotny M., Bulek K., Liu C., Li X., Hamilton T. (2011). Treatment with IL-17 prolongs the half-life of chemokine CXCL1 mRNA via the adaptor TRAF5 and the splicing-regulatory factor SF2 (ASF). *Nature Immunology*.

[72] Datta S., Novotny M., Pavicic P. G. (2010). IL-17 regulates CXCL1 mRNA stability via an AUUUA/tristetraprolin-independent sequence. *Journal of Immunology*.

[73] Zhang Y., Chandra V., Sanchez E. R. (2020). Interleukin-17–induced neutrophil extracellular traps mediate resistance to checkpoint blockade in pancreatic cancer. *Journal of Experimental Medicine*.

[74] Barnes B. J., Adrover J. M., Baxter-Stoltzfus A. (2020). Targeting potential drivers of COVID-19: neutrophil extracelluar traps. *Journal of Experimental Medicine*.

[75] Minns D., Smith K. J., Alessandrini V. (2021). The neutrophil antimicrobial peptide cathelicidin promotes Th17 differentiation. *Nature Communications*.

[76] Jiang L., Fang M., Tao R., Yong X., Wu T. (2020). Recombinant human interleukin 17A enhances the anti-*Candida* effect of human oral mucosal epithelial cells by inhibiting Candida albicans growth and inducing antimicrobial peptides secretion. *Journal of Oral Pathology & Medicine*.

[77] Furue M., Furue K., Tsuji G., Nakahara T. (2020). Interleukin-17A and keratinocytes in psoriasis. *International Journal of Molecular Sciences*.

[78] Dixon B. R. E. A., Radin J. N., Piazuelo M. B., Contreras D. C., Algood H. M. S. (2016). IL-17a and IL-22 induce expression of antimicrobials in gastrointestinal epithelial cells and may contribute to epithelial cell defense against *Helicobacter pylori*. *PLOS ONE*.

[79] Aujla S. J., Chan Y. R., Zheng M. (2008). IL-22 mediates mucosal host defense against Gram-negative bacterial pneumonia. *Nature Medicine*.

[80] Rabeony H., Petit-Paris I., Garnier J. (2014). Inhibition of keratinocyte differentiation by the synergistic effect of IL-17A, IL-22, IL-1*α*, TNF*α* and Oncostatin M. *PLoS ONE*.

[81] Guilloteau K., Paris I., Pedretti N. (2010). Skin inflammation induced by the synergistic action of IL-17A, IL-22, oncostatin M, IL-1*α*, and TNF-*α* recapitulates some features of psoriasis. *Journal of Immunology*.

[82] Ivanov I. I., Atarashi K., Manel N. (2009). Induction of intestinal Th17 cells by segmented filamentous bacteria. *Cell*.

[83] Schnell A., Littman D. R., Kuchroo V. K. (2023). T_H_17 cell heterogeneity and its role in tissue inflammation. *Nature Immunology*.

[84] Thakore P. I., Schnell A., Zhao M. (2022). The chromatin landscape of Th17 cells reveals mechanisms of diversification of regulatory and pro-inflammatory states. *bioRxiv*.

[85] Lai Y., Li D., Li C. (2012). The antimicrobial protein REG3A regulates keratinocyte proliferation and differentiation after skin injury. *Immunity*.

[86] Zhou X., Chen Y., Cui L., Shi Y., Guo C. (2022). Advances in the pathogenesis of psoriasis: from keratinocyte perspective. *Cell Death & Disease*.

[87] Kotla N. G., Rochev Y. (2023). IBD disease-modifying therapies: insights from emerging therapeutics. *Trends in Molecular Medicine*.

[88] Ogawa A., Andoh A., Araki Y., Bamba T., Fujiyama Y. (2004). Neutralization of interleukin-17 aggravates dextran sulfate sodium-induced colitis in mice. *Clinical Immunology*.

[89] Turchinovich G., Hayday A. C. (2011). Skint-1 identifies a common molecular mechanism for the development of interferon-*γ*-secreting versus interleukin-17-secreting *γδ* T cells. *Immunity*.

[90] Lee J. S., Tato C. M., Joyce-Shaikh B. (2015). Interleukin-23-independent IL-17 production regulates intestinal epithelial permeability. *Immunity*.

[91] Maxwell J. R., Zhang Y., Brown W. A. (2015). Differential roles for interleukin-23 and interleukin-17 in intestinal immunoregulation. *Immunity*.

[92] Kinugasa T., Sakaguchi T., Gu X., Reinecker H.-C. (2000). Claudins regulate the intestinal barrier in response to immune mediators. *Gastroenterology*.

[93] Ramani K., Garg A. V., Jawale C. V. (2016). The Kallikrein–Kinin system: a novel mediator of IL-17-driven anti-*Candida* immunity in the kidney. *PLOS Pathogens*.

[94] Wang J., Ding X. (2022). IL-17 signaling in skin repair: safeguarding metabolic adaptation of wound epithelial cells. *Signal Transduction and Targeted Therapy*.

[95] Zhang C.-J., Wang C., Jiang M. (2018). Act1 is a negative regulator in T and B cells via direct inhibition of STAT3. *Nature Communications*.

[96] Majumder S., Amatya N., Revu S. (2019). IL-17 metabolically reprograms activated fibroblastic reticular cells for proliferation and survival. *Nature Immunology*.

[97] Konieczny P., Xing Y., Sidhu I. (2022). Interleukin-17 governs hypoxic adaptation of injured epithelium. *Science*.

[98] Brigas H. C., Ribeiro M., Coelho J. E. (2021). IL-17 triggers the onset of cognitive and synaptic deficits in early stages of Alzheimer’s disease. *Cell Reports*.

[99] Machhi J., Yeapuri P., Lu Y. (2021). CD4^+^ effector T cells accelerate Alzheimer’s disease in mice. *Journal of Neuroinflammation*.

